# Color It Real: A Program to Increase Condom Use and Reduce Substance Abuse and Perceived Stress

**DOI:** 10.3390/ijerph13010051

**Published:** 2015-12-22

**Authors:** Tiffany Zellner, Jennie Trotter, Shelia Lenoir, Kelvin Walston, L’dia Men-Na’a, Tabia Henry-Akintobi, Assia Miller

**Affiliations:** 1 Department of Community Health and Preventive Medicine, Morehouse School of Medicine Prevention Research Center, 720 Westview Drive SW, Atlanta, GA 30310, USA; takintobi@msm.edu; 2 Wholistic Stress Control Institute, Incorporated, 2545 Benjamin E Mays Drive, Atlanta, GA 30311, USA; jt@wholistic1.com (J.T.); skl@wholistic1.com (S.L.); kelvinwalston@hotmail.com (K.W.); ldiamennaa@aol.com (L.M.-N.); 3 McKing Consulting Corporation, 2900 Chamblee Tucker Road, Building 10, Suite 100, Atlanta, GA 30341, USA; amiller@msm.edu

**Keywords:** African Americans, young adults, substance abuse, HIV prevention, stress

## Abstract

Few interventions have targeted perceived stress as a co-occurring construct central to substance use and subsequent HIV/AIDS risk reduction among African American urban young adults. The Color It Real Program was a seven session, weekly administered age-specific and culturally-tailored intervention designed to provide substance abuse and HIV education and reduce perceived stress among African Americans ages 18 to 24 in Atlanta, GA. Effectiveness was assessed through a quasi-experimental study design that consisted of intervention (*n* = 122) and comparison (*n* = 70) groups completing a pre- and post-intervention survey. A series of Analysis of Variance (ANOVA) tests were used to assess pre- to post-intervention changes between study groups. For intervention participants, perceived stress levels were significantly reduced by the end of the intervention (*t*(70) = 2.38, *p* = 0.020), condom use at last sexual encounter significantly increased (*F* = 4.43, *p* = 0.0360), intervention participants were significantly less likely to drink five or more alcoholic drinks in one sitting (*F* = 5.10, *p* = 0.0245), and to use clean needles when injecting the drug (*F* = 36.99, *p* = 0.0001). This study is among the first of its kind to incorporate stress management as an integral approach to HIV/SA prevention. The program has implications for the design of other community-based, holistic approaches to addressing substance use and risky behaviors for young adults.

## 1. Introduction

Substance abuse by young adults often leads to risky behavior and adverse health outcomes. Substance use increases the likelihood of high risk sexual behaviors such as not using condoms, particularly when combined with illicit drugs and alcohol use [1]. This risky mix is found to be the most causal risk factor for transmission of HIV/AIDS among African American young adults [2,3]. Risky sexual behavior includes the lack of condom use during sex, unplanned sex, lack of perceived risk or awareness, lack of testing, multiple partners, and alcohol and substance abuse [4,5,6,7]. Common risk factors of substance abuse and HIV risk include chronic stress exposure [8] and community characteristics such as residential segregation, poverty, income inequality, and high unemployment [9,10].

The likelihood of risky behavior and substance abuse in young adults is associated with poor coping and stress management. Anthenelli’s [11] census of the association between stress and alcohol use demonstrated that drinking has the ability to both relieve stress and to be the cause of it, and that the effects of alcohol consumption are based on individual biology, genetic and environmental factors. According to Keyes, Hatzenbuehler, Grant and Hasin [12], the relationship between stress and substance abuse may be attributable to “minority” stress, among other factors. Both perceptions and objective indicators of discrimination are associated with alcohol use among racial/ethnic and sexual minorities. These observations demonstrate that exposure to stress in many forms is related to subsequent alcohol consumption and other drug use. Discrimination has been a well-documented determinant of the marginalization of ethnic minority and low socioeconomic status groups [13]. The stressors that result from individually experienced and institutionally sanctioned discriminatory practices are important to discussions of health disparities. Additional culture-specific factors that may influence the gap between the knowledge gained through behavioral health interventions and behaviors among African American young adults include the gender ratio imbalance of men to women, pressure on women to engage in risky sex in order to maintain relationships, perceived disproval of homosexuality by peers, and high rates of childhood sexual abuse among women, all of which increase vulnerability to engage in risky sexual behaviors [14,15,16,17,18,19].

While a few interventions have been found to be effective in reducing substance abuse and related co-morbidities among African American young adults [20,21,22,23] no identified studies target perceived stress as a co-occurring construct central to both HIV and substance use risk reduction among African American young adults. Existing STI behavioral interventions for African Americans have had only a modest impact in eliminating the HIV burden [15]. Strategies targeting HIV risk reduction among African American young adults have also tended to utilize single-gender interventions [16,20,22], focusing on young Black men who have sex with men [18], or college students only [17]. Most studies targeting African American young adults and college students have investigated risk behaviors without implementing an intervention [16,17,24,25]. Due to the high rates of HIV infection among young African Americans and the dearth of interventions targeting this group, there is a need for substance use behavioral interventions that incorporate stress management skills targeting African American young adults.

The purpose of this study is to evaluate the effectiveness of the Color It Real program in increasing condom use, and reducing substance abuse and perceived stress levels among African American males and females, ages 18–24 between September 2011 and October 2012. It is our hypothesis that participants in the Color it Real program will self-report an increase in condom use, and a reduction in substance abuse and perceived stress levels upon completion of the program.

### The Intervention

In response to the aforementioned health and behavioral intervention disparities, The Wholistic Stress Control Institute Inc. (WSCI) developed and implemented The Color it Real Intervention (Color it Real) for African American young adults ages 18–24. Color it Real includes seven two-hour HIV/substance use prevention sessions, outreach and recruitment services, peer education, life skills development, social media, and referral services for screening, testing, counseling and treatment. The program emphasizes access to health resources, making condoms available, training young adults on personal risks and stress reduction. Trained staff are central to supporting the young adults in changing their behavior and risk perceptions. In small groups, participants discuss the following topics: HIV/STI risk knowledge, assessing personal risk and avoiding sexual risk, the correct use of male and female condoms, identifying and managing triggers for unsafe sex, substance use and problem solving. Activities to promote positive attitudes and build effective communication included games, exercises, and role-playing. The theories that link program activities of the Color It Real Program and expected outcomes are the Self-efficacy Model [26,27], the Theory of Change Model [28,29], and the Health Belief Model [30]. The Self-Efficacy Model emphasizes how an individual may personalize social situations and instructions through selection, affective, motivational and cognitive processes. The incorporation of situational exercises and role play into Color it Real relate to this theory. Participants were able to view or reenact social scenarios, such as condom negotiation, and peer pressure to engage in substance use, then dialogue about appropriate responses to each situation and practice delivering their response, so that they would gain confidence if faced with a similar scenario in their lives. The Theory of Change is a tool for developing solutions to complex social problems. The theory included the assumptions about the process through which change will occur and specified the ways in which all of the required early and intermediate outcomes related to achieving the desired long-term change will be brought about and documented as they occur. The Health Belief Model provides the basis for other unique features incorporated into Color it Real designed to actively engage participants in personal health decision-making to inspire a sense of responsibility for their behavior. This model suggests that people’s beliefs about health problems, perceived benefits of action and barriers to action and self-efficacy explain engagement (or lack of engagement) in health-promoting behavior.

## 2. Experimental Section

### 2.1. Design

A quasi-experimental research design with nonequivalent groups was utilized for this study. The study protocol, design and intervention were reviewed and approved by the Morehouse School of Medicine Institutional Review Board prior to implementation.

### 2.2. Participants and Procedures

Eligibility criteria for participation in the study included residence in Fulton, Dekalb, Cobb or Clayton counties of Georgia, and self-identification as an African American age 18–24 years old. WSCI health educators utilized a purposive non-probability sampling plan from community and college sites which allowed for successful recruitment of participants fitting the racial/ethnic and age inclusion criteria. Recruitment events were held on college campuses and community organizations. Participants were recruited through face to face engagement with WSCI health educators and staff. If individuals met the eligibility criteria, they were offered the opportunity to be enrolled. Written informed consent was obtained from all participants.

WSCI staff also identified sites where the study would be conducted. Sites located in the target counties that serve high-risk populations with the racial/ethnic background and age range were specified for this study. There were six intervention and three comparison sites. Multiple sites were utilized for recruitment and retention purposes; by providing locations to receive the program that were in the participants’ community and convenient for them to attend would foster completion of the program. Participants at intervention group sites received the Color it Real intervention. Participants at comparison group sites only received generalized health information.

### 2.3. Instruments

Evaluation objectives were assessed through three survey instruments. The Substance Abuse and Mental Health Services Administration’s (SAMHSA) National Minority Substance Abuse and HIV Prevention Initiative 118-item survey [31] was completed by participants to assess demographic characteristics, attitudes and knowledge of substance abuse and HIV prevention, and sexual risk behavior. Participants also completed a supplemental 23-item Street Smart Evidence-Based Intervention Survey [32], which measured participant condom use and intentions. To assess stress level, the 14-item Perceived Stress Scale (PSS) was administered [33].

The outcome domains analyzed for intervention efficacy are:

*Substance Abuse Behavioral Intentions.* Substance use risky behaviors were defined by questions on alcohol consumption, tobacco and drug use intentions and risk perception. Participants responded to four questions on their likelihood to use substances in the next six months. Questions were measured on a scale from 1 (“Not at all likely”) to 4 (“Very likely”) to identify their intentions.

*Condom Use and Intentions.* Participants were asked four questions about their recent use of, and intention to use both male and female condoms. Response options included 1 (“Yes”), 0 (“No”).

*Perceived Stress*. This scale measures how unpredictable, uncontrollable life events are perceived by respondents as well as their current level of experienced stress. Participants responded to each of 14 questions using a Likert type 5-point scale (ranging from 0 = never to 5 = very often) to indicate how often they had the thoughts and feelings during the last month. Reverse-scoring was used for positively stated questions. These answers were summed to form a perceived stress scale composite score with a maximum scale score of 70.

### 2.4. Analysis

Matched pre- and post-surveys were utilized for analysis. Survey responses were analyzed, comparing frequencies and means for each outcome variable between intervention and comparison groups. We used paired t-tests to compare the means for the perceived stress score. We applied Analysis of Variance (ANOVA) in the Generalized Linear Model to examine the effect of the intervention. Our outcome variables were substance abuse behavioral intentions, condom use and intentions, and perceived stress. Before analyzing the data, we checked the normality of the outcome domains. The distribution of the outcome domains were close to normal distribution. A series of ANOVA, randomized block design, was conducted to determine if there was any difference in outcomes between intervention and comparison groups. The model was adjusted for potential covariates such as time to collect data (pretest, posttest), gender (male, female) and education (did not complete high school, completed high school, some college and above). We reported mean, standard deviation, F test statistics and P value. A *p*-value of <0.05 was used to signify statistical significance. Statistical analyses were carried out using SAS version 9.2 (SAS Institute Inc., Cary, NC, USA).

## 3. Results

Our total sample size comprised of 192 participants who completed pre-test and post-test surveys—122 participants in intervention group and 70 participants in comparison group. Table 1 provides detailed information on the characteristics of participants based on matched pre-test surveys. With respect to the intervention group, the average age of participants was 20 years old. One hundred and eighteen (96.7%) respondents classified themselves as African American and 99 (81.1%) identified themselves as female. Almost one-third (34.4%) completed high school, 39.3% did not complete high school, 26.2% had some college education and had some postgraduate study. Although there was a difference between the intervention and comparison groups with respect to sample size, gender and education, differences for primary inclusion criteria, including age and race, were not statistically significant, implying that there was no impact of sample size on the results.

### 3.1. Substance Abuse

Table 2 displays results of the analysis related to changes in behavioral intentions associated with substance abuse. For the question related to the intention “To drink five or more alcoholic drinks in one sitting” there was a statistically significant difference between intervention and comparison groups (*F* = 5.10, *p* = 0.0245), indicating a positive impact of the program based on this indicator. Similar results were found for the use of clean needles when injecting drugs, with a statistically significant difference between the intervention and comparison groups (*F* = 36.99, *p* = 0.0001).

**Table 1:** Baseline characteristics between study groups.

Characteristic		Total *n* = 192	Intervention Group *n* = 122	Comparison Group *n* = 70	Group Difference *p*-Value
Age	Mean age	20.4	20.4	20.3	0.3516
		*n* (%)	*n* (%)	*n* (%)	
Gender	Male	49 (25.7%)	22 (18.0%)	27 (38.6%)	0.0019 *
	Female	142 (74.4%)	99 (81.1%)	43 (61.4%)	
Race	Black/African American	181 (94.3%)	118 (96.7%)	63 (90.0%)	0.1936
	White	4 (2.1%)	1 (0.8%)	3 (4.3%)	
	Hispanic/Latino	4 (2.1%)	1 (0.8%)	3 (4.3%)	
	Asian	1 (0.52%)	1 (0.8%)	0 (0.0%)	
	Other	2 (1.04%)	1 (0.8%)	1 (1.4%)	
Education	Did not complete high school	63 (32.8)	48 (39.3%)	15 (21.4%)	0.0004 *
	Completed high school	59 (30.7)	42 (34.4%)	17 (24.3%)	
	Some college and higher	70 (36.5)	32 (26.2%)	38 (54.3%)	

* Significant at *p*-value < 0.05.

**Table 2:** Results of ANOVA (Generalized Linear Model) Difference of Intervention *versus* Comparison Group in Substance Abuse Behavioral Intentions.

Question In the Next 6 Months, How Likely Are You to…?	Group	Pre-Test Mean (SD)	Post-Test Mean (SD)	Intervention Group *vs.* Comparison Group	
				F-Statistic	*p*-Value **
To drink five or more alcoholic drinks in one sitting?	Intervention	1.59 (0.95)	1.49 (0.94)	**5.10**	0.0245 *
	Comparison	1.84 (1.17)	2.14 (1.29)		
To use any illegal drugs (including prescription drugs) to get high?	Intervention	1.33 (0.83)	1.40 (0.93)	1.77	0.1836
	Comparison	1.72 (1.15)	1.36 (0.72)		
To use injection drugs without a doctor’s orders, just to feel good or to get high?	Intervention	1.06 (0.35)	1.10 (0.54)	1.16	0.2820
	Comparison	1.30 (0.87)	1.10 (0.51)		
To use clean needles when injecting drugs?	Intervention	2.20 (1.64)	3.00 (1.73)	**36.99**	0.0001 *
	Comparison	2.75 (1.54)	3.00 (1.41)		

* Significant at *p*-value < 0.05; ** Adjusted for gender and education.

### 3.2. Sexual Behavior and Condom Use Intention

There were increases in the favorable direction for all questions related to condom use intention for intervention group participants (see Table 3). Comparison of study groups indicates a statistically significant difference with a greater proportion of intervention participants demonstrating use of a condom at last sexual encounter (*F* = 4.43, *p* = 0.0360) and intention to use a female condom in the next 3 months at post-test survey (*F* = 8.66, *p* = 0.0035).

**Table 3:** Results of Analysis of Variance (Generalized Linear Model) of Difference of Intervention *versus* Comparison Group in Condom Usage.

Question	Group	Pre-test Mean (SD)	Post-test Mean (SD)	Intervention Group *vs.* Comparison Group	
				F-Statistic	*p*-Value **
The last time you had sex did you use a condom?	Intervention	0.63(0.49)	0.75(0.44)	**4.43**	0.0360 *
	Comparison	0.61(0.49)	0.60(0.49)		
The next time do you plan to use a condom?	Intervention	0.86(0.34)	0.90(0.31)	0.11	0.7398
	Comparison	0.85(0.36)	0.88(0.33)		
In the next 3 months do you plan to use a condom when you have sex?	Intervention	0.86(0.34)	0.90(0.30)	0.13	0.7223
	Comparison	0.86(0.35)	0.87(0.34)		
In the next 3 months do you plan to use a female condom?	Intervention	0.30(0.46)	0.37(0.48)	**8.66**	0.0035 *
	Comparison	0.13(0.33)	0.17(0.38)		

* Significant at *p*-value < 0.05; ** Adjusted for gender and education.

### 3.3. Perceived Stress

**Figure 1:**
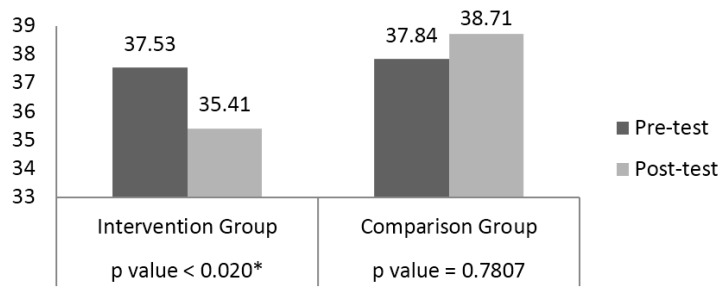
Perceived Stress Scale Composite Score for Intervention and Comparison Groups.

Figure 1 displays results of the analysis related to changes in perceived stress level. Prior to the program, the mean perceived stress score for the intervention group was 37.53 and after the program the mean score was 35.41, representing a statistically significant mean decrease for the intervention group (*t*(70) = 2.38, *p* = 0.020). The comparison group showed an increase of mean perceived stress score from pre- to post-test of 37.84 to 38.71, respectively. Comparison group scores were not statistically significant from pre- to post-test (*t*(48) = −0.28, *p* = 0.7807).

Of the 14 questions in the PSS, there were significant differences between intervention and comparison study groups for several outcome variables (Table 4). There was a statistically significant difference between the intervention and comparison group in answers to questions related to feeling confident about their ability to handle personal problems (*p* = 0.0006), being able to control irritations in life (*p* = 0.0240), feeling on top of things (*p* = 0.0049) and feeling calm and grounded (*p* = 0.0032). These results demonstrate positive effects of Color it Real on the intervention group.

**Table 4:** Results of Generalized Linear Model Analysis of Group Differences in Perceived Stress Scale Scores.

Question In the Last Month, How Often Have You…	Group	Pre-Test Mean (SD)	Post-Test Mean (SD)	Intervention *vs.* Comparison Group	
				F-Statistic	*p*-Value ^**^
Been upset because of something that happened unexpectedly?	Intervention	3.2 (1.1)	3.0 (1.1)	0.03	0.8726
	Comparison	3.0 (1.3)	2.9 (1.1)		
Felt that you were unable to control the important things in your life?	Intervention	2.9 (1.2)	2.5 (1.3)	0.03	0.8638
	Comparison	2.7 (1.2)	2.8 (1.1)		
Felt nervous and stressed?	Intervention	3.3 (1.2)	3.0 (1.2)	0.89	0.3456
	Comparison	3.3 (1.2)	2.9 (1.1)		
Felt confident about your ability to handle your personal problems?	Intervention	2.1 (1.1)	2.0 (1.1)	**11.98**	0.0006 *
	Comparison	2.4 (1.2)	2.6 (1.2)		
Felt that things were going your way?	Intervention	2.7 (1.0)	2.4 (1.1)	2.30	0.1306
	Comparison	2.7 (1.0)	2.8 (1.1)		
Found that you could not cope with all the things that you had to do?	Intervention	2.9 (1.1)	2.5 (1.1)	1.62	0.2042
	Comparison	2.8 (1.1)	2.8 (1.2)		
Been able to control irritations in your life?	Intervention	2.5 (1.1)	2.3 (1.1)	**5.15**	0.0240 *
	Comparison	2.5 (1.1)	3.0 (1.1)		
Felt that you were on top of things?	Intervention	2.3 (1.0)	2.1 (1.0)	**8.06**	**0.0049**
	Comparison	2.4 (1.0)	2.8 (1.1)		
In the last month, how often have you been angered because of things that happened that were outside of your control?	Intervention	3.0 (1.2)	2.7 (1.1)	0.88	0.3483
	Comparison	2.9 (1.3)	2.8 (1.0)		
Felt difficulties were piling up so high that you could not overcome them?	Intervention	2.6 (1.2)	2.5 (1.3)	3.24	0.0728
	Comparison	2.8 (1.2)	2.7 (1.1)		
Been able to overcome being upset to handle something unexpectedly?	Intervention	2.6 (1.2)	2.5 (1.2)	2.80	0.0956
	Comparison	2.8 (1.1)	2.8 (1.1)		
Felt calm and grounded?	Intervention	2.2 (1.0)	2.2 (1.2)	**8.87**	0.0032 *
	Comparison	2.4 (1.1)	2.6 (1.1)		
Found that you were coping well with all the things that you had to do?	Intervention	2.4 (1.0)	2.2 (1.1)	2.90	0.0894
	Comparison	2.4 (1.2)	2.7 (1.1)		
Had challenges that were piling up so high and that you were able to overcome them?	Intervention	3.0 (1.2)	3.3 (1.2)	0.01	0.9426
	Comparison	3.1 (1.2)	2.9 (1.2)		

* Significant at *p*-value < 0.05; ** Adjusted for gender and education.

## 4. Discussion

Analyses conducted to evaluate the intervention effect of Color it Real indicated a more favorable change in substance abuse behavioral intentions, condom use and intentions, and perceived stress level among those who participated in the intervention program than the comparison group. The intervention group showed statistically significant increases in behavioral intentions towards decreased alcohol use. They also demonstrated a significant increase in the intention to use clean needles. Intervention participants also significantly improved in condom use at last sexual encounter, and their intention to use a female condom. Study findings for perceived stress also demonstrated Color It Real’s significantly positive effect among intervention participants, when compared to the comparison group. Intervention participants’ feelings of confidence about abilities to handle personal problems, and being on top of things were all significantly more improved by the end of the intervention. While several articles [34,35] have explored the relationships between coping and HIV risk behaviors, there is little consensus on the impact of HIV intervention models on life stressors. This study identified decreased perceptions of stress among intervention participants, which included males, females, community dwelling and college-enrolled participants, thereby supporting the effective reach of the program to multiple audiences.

### 4.1. Strengths

Beyond the positive implications of results described above, additional strengths of this study are noteworthy. First, a satisfactory response rate to very sensitive and personal questions was observed. This is likely attributable to WSCI health educators who were socially and contextually congruent to participants. They also offered ongoing case management and relationship building to assuage participants’ concerns regarding disclosure of individual responses. Second, confidential survey administration allowed for pre-test and post-test matching by non-personal identifiers with results that were likely stronger than if anonymous survey administration had been implemented. Third, SAMHSA, Street Smart EBI and PSS surveys comprehensively addressed the important complexity of the intervention’s content. They were used to assess not only changes in substance abuse and HIV/AIDS knowledge, behavior and perception, but the ability to cope with stress. Finally, as noted in the literature, gender imbalance, power, and negotiation are critical elements of the risk reduction dynamic among African American males and females in heterosexual relationships. Therefore, the program’s focus on co-educational environments, with facilitation by both male and female health educators, contributed to the improved risk reduction behavioral intentions.

### 4.2. Limitations

Limitations of this study are also acknowledged. A rather small, purposive non-probability sample of program participants were recruited at local sites in Metropolitan Atlanta; therefore, the results are not generalizable to individuals that did not meet program inclusion criteria. However, the use of a comparison group provided robust data analysis to mitigate threats to validity and to identify intervention-specific outcomes. Due to the sensitive nature of some of the questions, some respondents may have provided socially-desirable answers or adjusted their responses. Finally, an extended follow-up period (9 to 12 months) may have facilitated observation of longer-term impact of the intervention rather than those associated with post-intervention results reported in this study.

### 4.3. Implications for Future Research

Color it Real’s results demonstrate important implications for future studies. In this study, individual level socioeconomic characteristics were similar among the intervention and comparison groups, but future studies should identify and control for community-level indices that may also impact outcomes. The ethnic disparities that persist for the majority of health outcomes in the United States demonstrate the need for expanded investigation of a more comprehensive array of variables that influence health outcomes at multiple levels. Results signal that public health interventions are more successful if culturally appropriate educational strategies are coupled with audience- relevant materials (*i.e.*, current videos, music, *etc.*) and case management approaches towards the adoption of risk reduction for substance use, decreased perceived stress and, ultimately, HIV/AIDS. Future studies may increase risk reduction effectiveness through approaches that address and measure multilevel issues, including perceived stress and self-efficacy in community contexts towards understanding of their roles on substance abuse and risky sexual behavior. In response to statistically significant differences by gender and education in adjusted models, future studies should address the differential impact of co-educational programs on sub-groups of African Americans aged 18–24. Expanded model exploration and theoretical framework testing to identify the most relevant constructs and pathways for transitioning knowledgeable African American young adults to improve behaviors are also recommended for future research studies. These factors are not static, but change with population demographic shifts and the political will to invest in not only individual, but community health. In summary, continued innovation and tailoring of strategies to reach young adults, particularly minority populations, is critical to shaping health outcomes.

## 5. Conclusions

This study is among the first of its kind to incorporate stress management as an integral approach to HIV/SA prevention. The Color it Real curriculum and peer-based facilitation effectively engaged participants, due to foci addressing both individual and contextual issues resonating with them as minority young adults in metropolitan, urban neighborhoods. The program has implications for the design of other community-based, holistic approaches to addressing substance use, perceived stress and risky behaviors for young adults.

## References

[1] CDC HIV Among Youth Fact Sheet; 2013. http://www.cdc.gov/hiv/group/age/youth/index.html.

[2] CDC HIV/AIDS Fact Sheet: HIV among African Americans; 2011. http://www.cdc.gov/hiv/group/racialethnic/africanamericans/.

[3] DCH Georgia HIV/AIDS Surveillance Summary: Data through 31 December 2009; 2010. http://www.georgiaaids.org/files/GA-HIV-AIDS_Surveillance-Summary.pdf.

[4] Carter M.W., Kraft J.M., Hatfield-Timajchy K., Hock-Long L., Hogben M. (2011). STD and HIV Testing Behaviors Among Black And Puerto Rican Young Adults. Perspect. Sex. Reprod. Health.

[5] Kennedy B.L., Roberts S.T. (2009). Truths and Myths That Influence the Sexual Decision-Making Process Among Young Multiethnic College Women. Arch. Psychiatr. Nurs..

[6] Kogan S.M., Brody G.H., Chen Y.F., Grange C.M., Slater L.M., di Clemente R.J. (2010). Risk and Protective Factors for Unprotected Intercourse Among Rural African American Young Adults. Public Health Rep..

[7] Randolph M.E., Torres H., Gore-Felton C., Lloyd B., McGarvey E.L. (2009). Alcohol Use and Sexual Risk Behavior among College Students: Understanding Gender and Ethnic Differences. Am. J. Drug Alcohol Abus..

[8] Enoch M.A. (2011). The role of early life stress as a predictor for alcohol and drug dependence. Psychopharmacology.

[9] Bluthenthal R., Do D.P., Finch B., Martinez A., Edlin B.R., Kral A.H. (2007). Community Characteristics Associated with HIV Risk among Injection Drug Users in the San Francisco Bay Area: A Multilevel Analysis. J. Urban Health.

[10] Lampinen T.M., Joo E., Seweryn S., Hershow R.C., Wiebel W. (1992). HIV seropositivity in community-recruited and drug treatment samples of injecting drug users. AIDS.

[11] Anthenelli R.M. (2012). Overview: Stress and alcohol use disorders revisited. Alcohol Res. Curr. Rev..

[12] Keyes K.M., Hatzenbuehler M.L., Grant B.F., Hasin D.S. (2012). Stress and Alcohol: Epidemiologic Evidence. Alcohol Res. Curr. Rev..

[13] Gee G.C. (2002). A Multilevel Analysis of the Relationship Between Institutional and Individual Racial Discrimination and Health Status. Am. J. Public Health.

[14] Adimora A.A., Schoenbach V.J. (2005). Social Context, Sexual Networks, and Racial Disparities in Rates of Sexually Transmitted Infections. J. Infect. Dis..

[15] Barrow R.Y., Berkel C., Brooks L.C., Groseclose S.L., Johnson D.B., Valentine J.A. (2008). Traditional Sexually Transmitted Disease Prevention and Control Strategies: Tailoring for African American Communities. Sex. Transm. Dis..

[16] Freeman C. (2010). The missing element: Incorporating culturally-specific clinical practices in HIV prevention programs for African-American females. J. Cult. Divers..

[17] Mongkuo M.Y., Mushi R.J., Thomas R. (2010). Perception of HIV/AIDS and socio-cognitive determinants of safe sex practices among college students attending a historically black college and university in the United States of America. J. AIDS HIV Res..

[18] Peterson J.L., Jones K.T. (2009). HIV Prevention for Black Men Who Have Sex With Men in the United States. Am. J. Public Health.

[19] Wyatt G.E. (2009). Enhancing Cultural and Contextual Intervention Strategies to Reduce HIV/AIDS Among African Americans. Am. J. Public Health.

[20] Baker J.L., Brawner B., Cederbaum J.A., White S., Davis Z.M., Brawner W., Jemmott L.S. (2012). Barbershops as venues to assess and intervene in HIV/STI risk among young, heterosexual African American men. Am. J. Men’s Health.

[21] Fisher H.H., Patel-Larson A., Green K., Shapatava E., Uhl G., Kalayil E.J., Moore A., Williams W., Chen B. (2011). Evaluation of an HIV prevention intervention for African Americans and Hispanics: Findings from the VOICES/VOCES community-based organization behavioral outcomes project. AIDS Behav..

[22] Kennedy S.B., Nolen S., Pan Z., Smith B., Applewhite J., Vanderhoff K.J. (2013). Effectiveness of a brief condom promotion program in reducing risky sexual behaviours among African American men. J. Eval. Clin. Pract..

[23] Latkin C.A., Sherman S., Knowlton A. (2003). HIV prevention among drug users: Outcome of a network-oriented peer outreach intervention. Health Psychol..

[24] Hou S.I. (2009). HIV-related behaviors among black students attending Historically Black Colleges and Universities (HBCUs) versus white students attending a traditionally white institution (TWI). AIDS Care.

[25] Rose M.S. (2008). African American college freshman students’ knowledge, attitudes, beliefs, and behaviors related to HIV: A preliminary investigation. Int. J. Allied Health Sci. Pract..

[26] Bandura A. (1977). Self-efficacy: Toward a unifying theory of behavioral change. Psychol. Rev..

[27] Bandura E.A. Self-Efficacy: The Exercise of Control. http://search.proquest.com/openview/55c56d1a75f8440c4bea93781b0dc952/1?pq-origsite=gscholar#top.

[28] Becker M.H. (1974). The Health Belief Model and Preventive Health Behavior. Health Educ. Monogr..

[29] Carpenter C.J. (2010). A meta-analysis of the effectiveness of health belief model variables in predicting behavior. Health Commun..

[30] Brest P. (2010). The power of theories of change. Stanf. Soc. Innov. Rev..

[31] SAMHSA National Minority SA/HIV Prevention Initiative, Cohort 6 Adult Questionnaire; 2008. http://www.samhsa.gov/grants/gpra-measurement-tools/csap-gpra.

[32] CDC Street Smart Evaluation Field Guide; 2008. https://effectiveinterventions.cdc.gov/Files/Street_Smart_Eval_Field_Guide_09-1023.pdf.

[33] Cohen S., Kamarck T., Mermelstein R. (1983). A global measure of perceived stress. J. Health Soc. Behav..

[34] Ickovics J.R., Beren S.E., Grigorenko E.L., Morrill A.C., Druley J.A., Rodin J. (2002). Pathways of risk: Race, social class, stress, and coping as factors predicting heterosexual risk behaviors for HIV among women. AIDS Behav..

[35] El-Bassel N., Caldeira N.A., Ruglass L.M., Gilbert L. (2009). Addressing the unique needs of African American women in HIV prevention. Am. J. Public Health.

